# The Growth Hormone Receptor: Mechanism of Receptor Activation, Cell Signaling, and Physiological Aspects

**DOI:** 10.3389/fendo.2018.00035

**Published:** 2018-02-13

**Authors:** Farhad Dehkhoda, Christine M. M. Lee, Johan Medina, Andrew J. Brooks

**Affiliations:** ^1^The University of Queensland Diamantina Institute, Translational Research Institute, The University of Queensland, Brisbane, QLD, Australia

**Keywords:** growth hormone, growth hormone receptor, Janus kinase 2, Src family kinase, insulin-like growth factor 1, suppressor of cytokine signaling

## Abstract

The growth hormone receptor (GHR), although most well known for regulating growth, has many other important biological functions including regulating metabolism and controlling physiological processes related to the hepatobiliary, cardiovascular, renal, gastrointestinal, and reproductive systems. In addition, growth hormone signaling is an important regulator of aging and plays a significant role in cancer development. Growth hormone activates the Janus kinase (JAK)–signal transducer and activator of transcription (STAT) signaling pathway, and recent studies have provided a new understanding of the mechanism of JAK2 activation by growth hormone binding to its receptor. JAK2 activation is required for growth hormone-mediated activation of STAT1, STAT3, and STAT5, and the negative regulation of JAK–STAT signaling comprises an important step in the control of this signaling pathway. The GHR also activates the Src family kinase signaling pathway independent of JAK2. This review covers the molecular mechanisms of GHR activation and signal transduction as well as the physiological consequences of growth hormone signaling.

## Introduction

**Figure 1:**
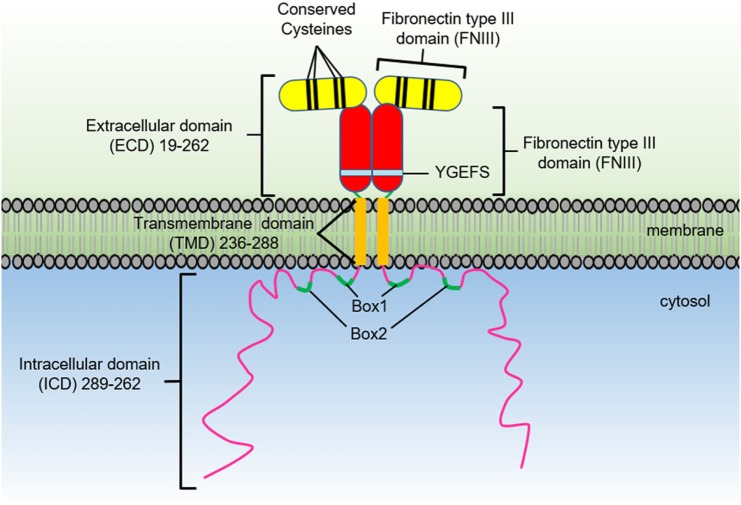
The growth hormone receptor domain organization.

**Figure 2:**
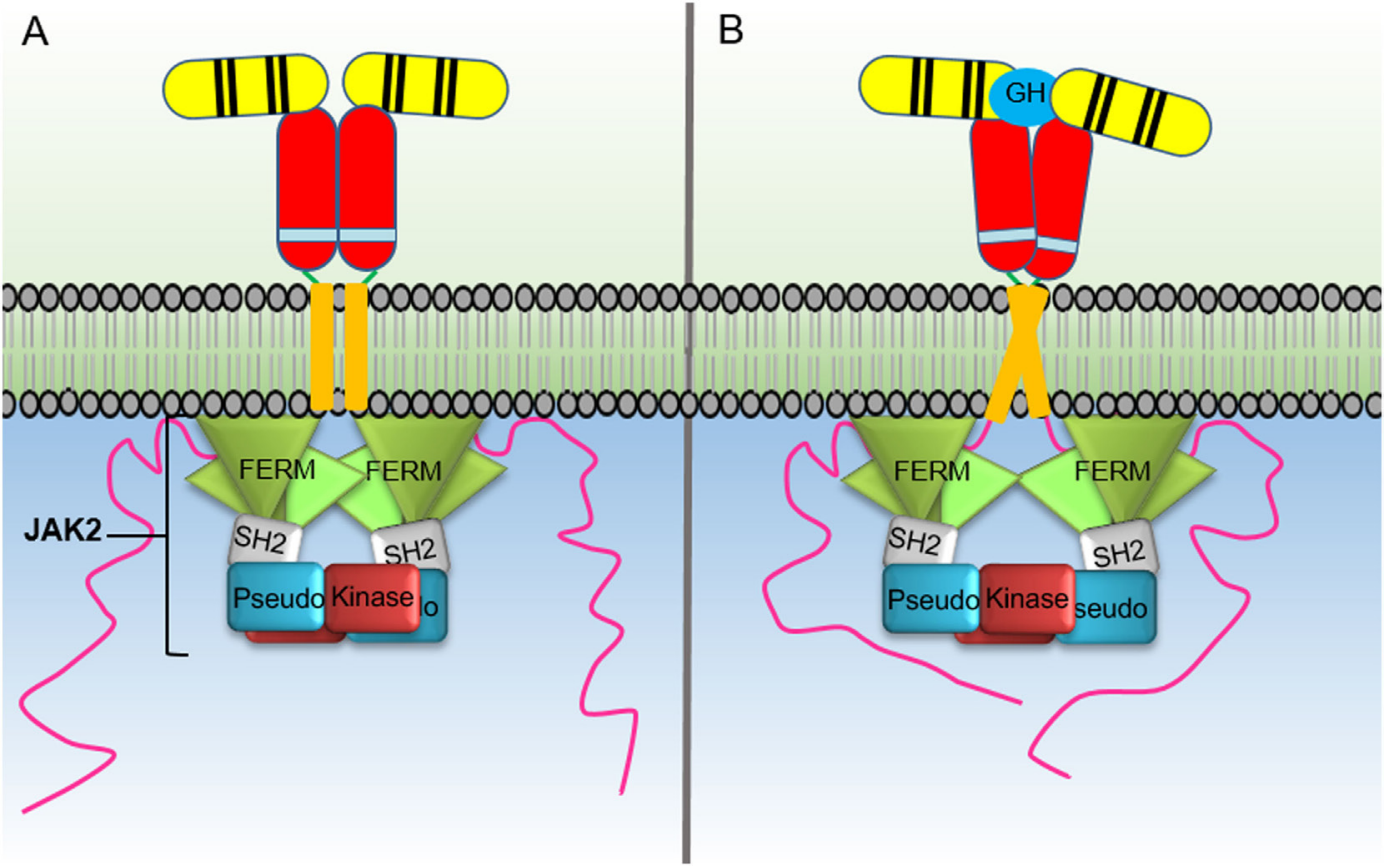
Activation of the growth hormone receptor (GHR) by growth hormone. In the inactive homodimeric GHR, **(A)** the Janus kinase (JAK) 2 kinase domain (KD) is inhibited in *trans* by interaction with the pseudokinase domain from the JAK2 bound to the opposing receptor within the homodimer. GH binding to the GHR extracellular domain **(B)** results in conformational changes that cause the transmembrane domains to transition from a parallel interaction to a left-handed crossover interaction. These structural changes cause a separation of the intracellular domains to the Box1 and Box2 motifs and the associated JAK2 molecules. The movement of the associated JAK2s dissociates the inhibitory interaction of the pseudokinase from the KD and brings the two JAK2 KDs in close proximity resulting in *trans* phosphorylation and activation.

**Figure 3:**
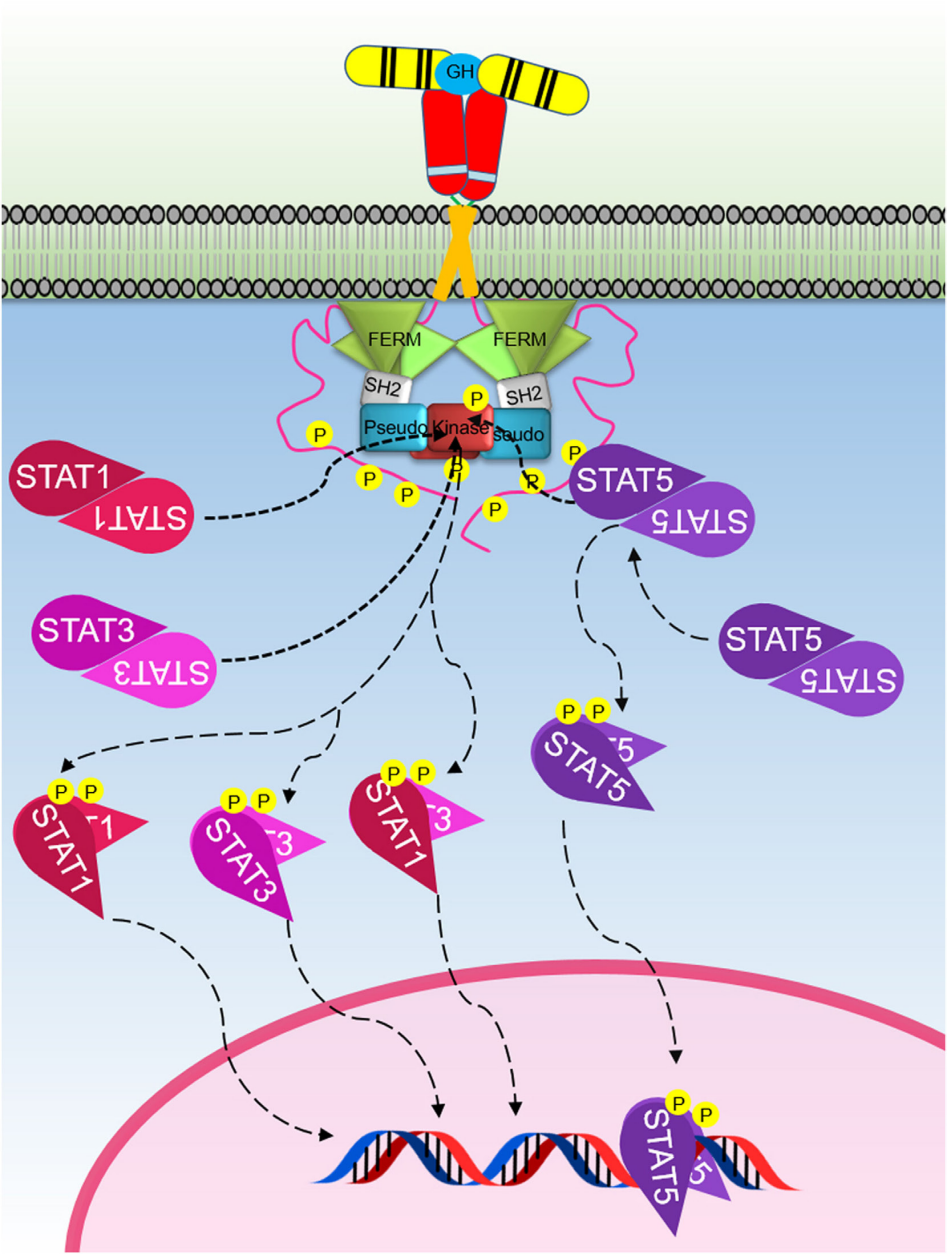
The Janus kinase (JAK)–signal transducer and activator of transcription (STAT) signaling pathway initiated by the activated growth hormone receptor. Activated JAK2 phosphorylates tyrosines on the intracellular domain of the receptor. Inactive-STAT5 dimers bind these phosphorylated tyrosine residues on the receptor, and the STAT5 is subsequently phosphorylated by JAK2 forming different active-STAT5 dimers that are translocated to the nucleus, bind DNA, and act as transcription factors. STAT1 and STAT3 are phosphorylated and activated by JAK2. Active STAT1 and STAT3 form homodimers or heterodimers, are translocated to the nucleus, bind DNA, and act as transcription factors.

The growth hormone receptor (GHR) is a member of the class I cytokine receptor family, which includes more than 30 receptors such as the prolactin receptor (PRLR), erythropoietin receptor (EPOR), thrombopoietin receptor (TPOR), granulocyte-macrophage colony-stimulating factor receptor, interleukin-3 receptor, interleukin-6 receptor, and interleukin-7 receptor (1, 2). GHR has been considered the archetypal class I cytokine receptor as it was the first cytokine receptor to be cloned and have its extracellular domain (ECD) crystal structure solved (3). GHR is a 638 amino acid long homodimeric receptor with one cytokine receptor homology domain (CRH), a single-pass transmembrane domain, and cytoplasmic intracellular domain (ICD) (Figure 1). With the exception of GHR, all members of the class I cytokine receptor family contain a WSXWS motif in the ECD. The WSXWS motif is important for expression and stability of the receptor and comprises a consensus sequence for *C*-mannosylation. For the IL-21R, the WSXWS has been shown to be mannosylated at the first tryptophan where the sugar chain appears to form structurally important interactions that bridge the two fibronectin domains (4, 5). GHR has in place of the WSXWS a similar sequence of YGeFS that has an analogous function in expression and stability of the receptor (1). Cytokine receptors lack an intrinsic protein tyrosine kinase (PTK) activity and therefore rely on binding non-receptor PTKs for their signal transduction. Within the ICD of all class I cytokine receptors is a proline-rich Box1 motif that is located a short distance from the cell membrane. A less conserved Box2 sequence consisting of acidic and aromatic residues is located a short distance C-terminal of the Box1 (1). The Box1 motif acts as a binding site for a cognate Janus kinase (JAK) of which there are four family members, JAK1, JAK2, JAK3, and TYK2 that can bind to specific receptors (Table 1). For GHR, the only JAK family member that binds the receptor is JAK2. GH binding to GHR results in activation of JAK2, which subsequently phosphorylates multiple tyrosine residues on the ICD of the receptor (Figure 2) (6, 7). This provides a scaffold for binding of STAT5a and STAT5b, which are subsequently phosphorylated by JAK2 upon receptor docking (Figure 3) (8). GHR also activates STAT1 and STAT3 *via* JAK2; however, these STATs do not appear to require binding to the phosphorylated receptor. Other signaling pathways such as the Ras/extracellular signal-regulated kinase (ERK) and PI 3-kinase/Akt are also activated by GHR (7, 9). The consequence of these GH-mediated cellular signaling pathways in a diverse range of cell types is responsible for the large range of physiological processes regulated by GH.

**Table 1:** Class I cytokine receptors and their Janus kinase (JAK)–signal transducer and activator of transcription (STAT) signaling partners.

Receptor group	Receptor	Partner JAK/s	Activated STAT/s	Reference
Homomeric receptors	Growth hormone receptor	JAK2	STAT1, STAT3, and STAT5	(10, 11)
	EPOR	JAK2	STAT1, STAT3, and STAT5	(12–14)
	Prolactin receptor	JAK2	STAT1, STAT3, and STAT5	(15–17)
	Thrombopoietin receptor	JAK2 and TYK2	STAT1, STAT3, and STAT5	(18–20)
	G-CSFR	JAK1 and JAK2	STAT1, STAT3, and STAT5	(21, 22)
	OBR	JAK2	STAT3, STAT5, and STAT6	(23, 24)

βc Heteromeric receptors	IL-3Rα	JAK2	STAT5 and STAT6	(25–27)
	IL-5Rα	JAK2	STAT1, STAT3, and STAT5	(28, 29)
	GMRα	JAK2	STAT1, STAT3, and STAT5	(30, 31)

GP130 heteromeric	IL-6Rα	JAK1, JAK2, and TYK2	STAT1 and STAT3	(32, 33)
	IL-11Rα	JAK1, JAK2, and TYK2	STAT1 and STAT3	(34, 35)
	LIFR	JAK1 and JAK2	STAT1 and STAT3	(36)

γc Heteromeric receptors	IL-2Rα	JAK1 and JAK3	STAT3 and STAT5	(37)
	IL-4Rα	JAK1 and JAK3	STAT6	(38, 39)
	IL-7Rα	JAK1 and JAK3	STAT3 and STAT5	(40)

*Example receptors shown for each receptor group*.

## GHR Structure and Activation Mechanism

The crystal structure of GH bound to the GHR ECD showed that one GH molecule bound two GHR molecules (3). Biophysical studies demonstrated that GH binds initially to a single receptor through its “site 1” motif and then subsequently binds to the second receptor *via* its “site 2” interactions (41, 42). The major energy contributor residues of the hormone–receptor complex were identified as residues in the receptor hydrophobic patch, where tryptophan^104^ and tryptophan^169^ contributed significantly to site 1 interactions. In addition, tryptophan^104^ plays a key role in the weaker site 2 interaction (43, 44). These studies suggested GH binding causes receptor dimerization, which induces intracellular signal transduction. This dimerization-induced activation model was supported by a study using a hybrid receptor of the GHR ECD fused to the base of the CRH domain of granulocyte colony-stimulating factor receptor (G-CSFR) with the remaining fibronectin type III homology domains (FNIII), transmembrane, and ICD. Monoclonal antibodies to the GHR ECD activated the hybrid receptor whereas monovalent fragments did not; however, the additional FNIII domains may have influenced these data (45). In addition, of eight antibodies that were effective agonists for the GHR/G-CSFR hybrid, only one antibody acted as an agonist with weak activity on full-length GHR, suggesting that receptor dimerization alone is not sufficient for activation (46). Later studies using site 2-modified GH antagonists, which would be predicted to bind only GHR monomer, appeared to bind GHR dimers expressed on cell membranes, thus demonstrating the existence of GHR dimers before GH binding (47). Subsequent studies confirmed that GHR dimers exist on the cell surface, and that dimers form in the endoplasmic reticulum. It was also shown that the extracellular region is not required for maintaining receptor dimers, although the lower FNIII domain may define receptor-dimer specificity (48–52). Data supporting the GHR preformed dimer conclusion were performed independently by more than one group using multiple techniques including co-immunoprecipitation, FRET, BRET, and fluorescence anisotropy (48–51). By analyzing the FRET efficiency with FRET reporters placed at the C-termini of full-length GHR and C-terminal truncations, it was found, in general, that the dimeric-GHR ICDs are positioned at a greater distance apart, the further they are from the transmembrane domain (50). This has been illustrated clearly for the closely related receptor PRLR where a recent full-length structure has been produced, which includes a molecular model of the ICD (53).

Crystallographic studies comparing the unliganded ECD of GHR to the ligand-bound form showed no major change in overall structure; however, a 7–9° of rotation between upper and lower FNIII-type domains of the CRH module was observed, which was small compared with the ligand-induced conformation change shown for the ECD of RTKs (50). It was also evident that ligand binding did not change FRET efficiency of GH receptors with N-terminal reporters confirming lack of major conformational change in the ECDs upon GH binding (50).

## Janus Kinase 2

Most members of the JAK family are widely expressed in many cell types, except JAK3 whose expression is restricted to cells of the hematopoietic lineage (54). While JAK1 and JAK2 are involved in diverse physiological actions such as hematopoiesis, immunity, development, and growth (1, 55, 56), JAK3 and Tyk2 are predominantly involved in homeostasis of immune system (57, 58).

Janus kinases are relatively large proteins ranging from 120 to 140 kD (~1,150 amino acids) (59). The N-terminal half of these proteins comprises N-terminal band 4.1, ezrin, radixin, moesin (FERM) domain followed by Src homology 2 (SH2) domain, which were primarily defined as Janus homology (JH) domains 3–7 (60). These domains play the major role in binding of JAKs to the cytoplasmic tails of their cognate cytokine receptors (61–63). The first evidence demonstrating the role of the N-terminus of JAK2 in receptor interactions was shown using JAK2 mutants, lacking the 239 N-terminal residues that rendered the JAK2 mutant incapable of binding GHR or the membrane proximal region of the common β chain (64, 65). This was further supported by studies showing that the FERM and SH2 domains of JAK1 are essential for association to oncostatin M receptor (OSMR), and that this binding is also required for upregulating surface expression of the OSMR (66, 67). Truncation studies showed that the membrane proximal 61 amino acids of GP130 are sufficient for signal transduction (68), and later it was shown for GHR that the membrane proximal 20 residues (which includes the Box1 motif) of the ICD is sufficient for JAK2 binding, but not sufficient for GH-induced activation of JAK2 (69). Until recently, little was known about the mechanism of receptor–JAK association or how JAKs gain specificity to their cognate receptors. Previous studies have shown that the Box1 motif, although required for JAK binding, is not sufficient to determine the specificity for a particular JAK to associate with its cytokine receptor, and that additional sequences in the receptor play an important role in specificity since JAK1 and JAK2 were shown to have different preferences for GHR, EPOR, and GP130 chimeras (70). The first crystal structure of a JAK bound to residues of a receptor ICD was shown for the FERM–SH2 of TYK2 in complex with the Box2 region of the interferon-α receptor 1 (IFNAR1) (71). This study revealed that the TYK2 FERM–SH2 domains form a clover shaped structure with the SH2 being the forth leaf. The structure showed the FERM domain has a canonical three-lobe structure (F1–F3), and the SH2 has a preserved SH2-like structure despite lacking the highly conserved arginine residue in the phosphotyrosine binding site (71, 72). Interestingly, the SH2 domain coordinates the interactions with IFNAR1 to form the initial connections with the receptor peptide in the C-terminus. Further insights regarding cytokine receptor recognition by JAKs emerged when the crystal structure of JAK1 bound to peptides derived from the IL-10 receptor (IL10RA) and interferon lambda receptor (IFNLR1) was solved (73). These studies showed the Box1 peptide bound to the F2 domain of the FERM while the Box2 peptide was not resolved in the structure. In addition, the binding kinetics of the IFNLR1 Box1 and Box2 showed that Box1 alone has a strong affinity while Box2 alone had no detectable interaction; however, the full Box1–Box2 peptide had a significantly enhanced affinity over Box1 alone, which was proposed to be due to the increased complex stability from the hydrophobic JAK1–Box2 interaction (73). Although neither of these recent structures showed simultaneous interactions between the JAK molecule and both Box1 and Box2 sequences, geometrically both sequences are capable of binding to single JAK protein (73, 74). The crystal structure of the JAK1 FERM–SH2 bound to peptides from IL10RA and IFNLR1 gave a provocative suggestion that the Box1 peptide bound to the FERM domain extends away from the FERM domain to interact with the SH2 domain of the neighboring JAK1 molecule in the crystal lattice. Further studies would be needed to determine if Box1–Box2 sequences can have relevant interactions with two JAKs simultaneously, although it was suggested that this may have been due to a crystal artifact (73). More recently, a crystal structure of the JAK1 FERM–SH2 has been solved with a continuous IFNLR1 Box1–Box2 peptide suggesting that lack full Box1–Box2 peptide in previous structures may have been a crystal artifact (75). Recently, a JAK2 FERM–SH2 structure has been solved; however, this structure is not in complex with any receptor sequence (76).

The C-terminal half of JAK proteins comprises a pseudokinase domain (PKD) followed by a kinase domain (KD), also known as JH2 and JH1, respectively (77). The KD is a typical tyrosine KD and is catalytically active, whereas the adjacent PKD is catalytically inactive and lacks critical residues despite containing a canonical kinase fold (78). The PKD is believed to regulate kinase activity, and its deletion in JAK2 and JAK3 was shown to increase basal activity of the kinase (64, 79–81). Biochemical studies have reported co-immunoprecipitation of JH1 and JH2 of JAK2, and that the pseudokinase acts to inhibit the kinase activity of JAKs (60, 79, 82). Furthermore, recent studies have shown that the PKD of JAK2, unlike the other members of the JAK family, exhibits some catalytic activity *via* autophosphorylation of two critical residues in the JH2–SH2 linker region, Ser^523^ and Tyr^570^, which play an important role in regulating the activity of the KD (82, 83).

## Mechanism of JAK2 Activation by the GHR

Recent studies have made major strides in elucidating the mechanism of JAK2 activation by GHR (84). Activation of the receptor by GH binding was found to result in an increase in distance between the Box2 motifs of the intracellular receptor domains. This revelation required a new model for JAK2 activation by cytokine binding whereby in the cytokine free receptor, the JAK2 KD is inhibited by a JAK2 PKD in *trans* (Figure 2). In this model, the KD from one JAK2 molecule bound to a Box1–Box2 motif from one receptor is inhibited by interacting with the PKD from a different JAK2 molecule bound to the opposing receptor within the receptor homodimer. Upon GH binding, there is a structural change in the ECD that changes the receptor transmembrane domain interaction from a parallel form to a left-handed crossover interaction. This structural transition leads to a separation of the ICD, at least to the Box1–Box2 motif, and dissociates the JAK2 kinase-pseudokinase *trans* interaction and brings the KDs in proximity allowing *trans* phosphorylation and activation (1, 51).

Currently, there is no crystal structure available for the JAK2 pseudokinase-kinase to allow observation of the interaction interface between each domain; however, crystal structures for the individual pseudokinase and KDs have been solved (83, 85). The pseudokinase-kinase crystal structure has presently been solved for only one JAK family member, Tyk2 (86). However, the functional Tyk2 interface was only inferred from the crystal structure as there were three significant potential interaction faces identified in the crystal lattice. It could not be determined directly in this structure as to how the pseudokinase and KDs were joined *via* the interdomain linker as this linker sequence was not resolved in the crystal structure. The potential dimer interface with the largest buried surface area was excluded from detailed analysis as the distance between the termini of each domain was too great to allow each domain to be linked by the interdomain linker peptide (86). However, for a *trans*-inhibition interaction model, there would be no requirement for each domain to be linked (87), therefore perhaps this interface with the largest buried surface area may have some biological relevance. The interface with the second largest buried surface area in the Tyk2 crystal lattice did place the C- and N-termini in close enough proximity to be linked by the interdomain linker, and this interface was investigated in detail and mutational data did support the biological significance of this interaction surface. There are a number of activating mutations identified for the JAK2 PKD; however, the location of these mutations suggests there may be two interaction interfaces that are involved in regulating the KD (88). For the Tyk2 pseudokinase-kinase crystal structure, a number of JAK-activating mutations were found to lie directly in the proposed domain interface while a second surface of activating mutations was found peripheral to the proposed dimer interface, which includes the position for the JAK2 V617F mutation commonly identified in myeloproliferative neoplasms (89). Interestingly, some JAK2-activating mutations including V617F have been shown to require interaction with a cytokine receptor for their activity, suggesting that these mutations only disrupt inhibition of the pseudokinase when the JAK2 is bound to a receptor (90–93). Intriguingly, this supports previous suggestions that the JAK2 pseudokinase interaction and inhibition of the KD is different between the receptor bound JAK2 and the receptor free JAK2 (90). Molecular modeling of the proposed JAK2 *trans*-inhibition interaction of the PKD with the KD placed the V617F in proximity to the activation loop of the KD; however, further direct functional and structural data supporting this interaction are currently not available. Although functional data supporting a *trans-*inhibition model were obtained when constitutive activation of JAK2 was shown with a JAK2 construct where the pseudokinase and KDs were swapped and this construct showed significant cytokine-independent activation only in the presence of wild-type JAK2 (51).

As the GHR ICD is intrinsically disordered with a number of transient secondary structures (94), the C-termini are likely a long distance apart in the inactivated receptor homodimer in a similar manner as illustrated in a recent full structural model of the PRLR (53). Studies using FRET reporters placed at the C-terminus of GHR have shown a transient increase in FRET shortly after GH addition (95). These data likely represent the flexible receptor ICD interacting with the activated JAK2 KDs and being phosphorylated upon multiple tyrosine residues, which subsequently interact with downstream signaling molecules and negative regulators of signaling.

## Signal Transducers and Activators of Transcription (STATs)

The STATs are important components of the signaling cascades triggered by various cytokines (Table 1). STATs were initially identified as cytokine-induced transcription factors in interferon treated cells (96–98). To date, seven different members of the STAT family have been identified which includes STAT1–4, 5a, 5b, and 6. STAT5a and 5b are encoded by two different genes with high-sequence homology, except for regions in the N- and C-termini (99). In addition, posttranslational modification and proteolytic cleavage have shown to produce additional forms of STAT1 and STAT3 (100). Although the STAT family members are activated *via* various cytokines, STAT2 and STAT6 are predominantly activated by IFN-α and IL-4, respectively (101). STAT proteins contain six domains, including an N-terminal domain, coiled coil domain, DNA-binding domain, linker domain, SH2 domain, and transactivation domain (98). Each STAT member can be activated by a JAK *via* phosphorylation of a conserved tyrosine. Activated STATs translocate to the nucleus where they act as transcriptional regulators. Although STAT members including STAT1, 3, and 5 are activated by JAK2 following GH induction (Figure 3), STAT5 is the predominant transcription factor that delivers GH-induced cell proliferation and actions. Activated STAT5 forms homodimers, whereas active STAT1 and STAT3 form both homodimers and STAT1–STAT3 heterodimers (Figure 3) (7, 102, 103).

## GHR-Mediated SRC Family Kinase (SFK) Signaling

Src family kinases are non-receptor tyrosine kinases, which comprise nine members, namely, Src, Lck, Hck, Fyn, Blk, Lyn, Fgr, Yes, and Yrk, all of which share a common multidomain topology consisting of consecutive SH3, SH2, and SH1 KDs and a “unique” region located close to the N-terminus, which differentiates each member of the family. The regulation of SFKs is accomplished through mechanisms involving phosphorylation, dephosphorylation, and complex intramolecular interactions that lead to SH3 and SH2 domain displacement (104, 105). Members of the SFKs are essential for the regulation of immune system function and cellular homeostasis (106). Lyn is a unique member of this family because it acts both as a signal amplifier and also as a signal inhibitor while others are known to only play a single role (107). The major functions of Lyn have been attributed to signaling of hematopoietic growth factor receptors and blood cell development as it is expressed in all blood cells except T lymphocytes (108).

**Figure 4:**
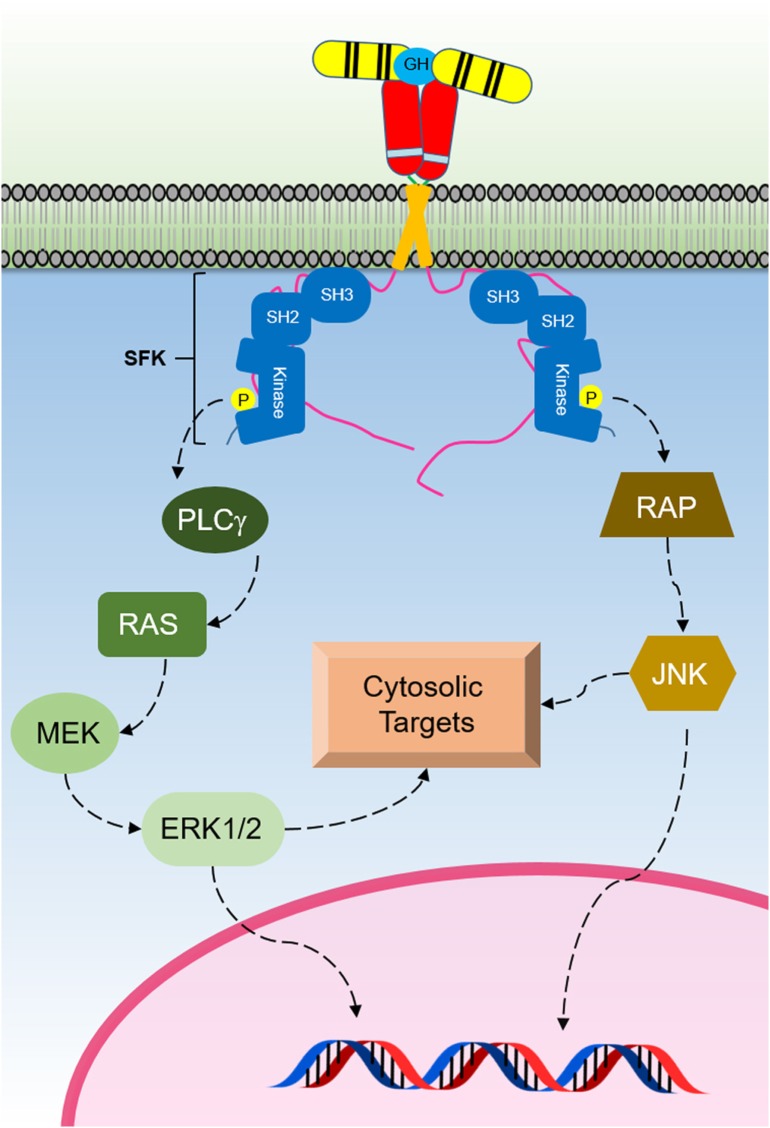
The Src family kinase (SFK) signaling initiated by the activated growth hormone receptor (GHR). SFKs are activated by GH binding to GHR. This signaling pathway activates ERK1/2 that regulates cytosolic targets and gene transcription.

Studies have shown that GH can activate SFKs independent of any JAK2 activation (Figure 4) (109). The SFK member Lyn was found to be associated with the membrane proximal 150 residues of the cytoplasmic domain of human GHR, and Lyn was still shown to be activated by GH when using JAK2-deficient γ2A cells. Furthermore, it was reported that a mutant of the GHR Box1, resulting in loss of GHR-mediated JAK2–STAT signaling, still responded to GH stimulation through the activation of SFKs and ERK1/2 (109, 110). These studies suggested that this novel SFK-dependent pathway involves the activation of PLCγ, and a subsequent release of inositol 1,4,5-triphosphate (IP_3_) and diacylglycerol which leads to an increase in the cytoplasmic calcium ion concentration-activating Ras guanine nucleotide exchange factor RasGRP1. This process results in RAS activation and initiation of the ERK1/2 signaling pathway (109). Related studies using growth hormone as a ligand confirmed that this JAK2-independent pathway can activate ERK1/2 through the activation of Ras-like small GTPases, RalA, and RalB in NIH-3T3 cells and through the activation of Rap1 and Rap2 (111, 112). Mutations introduced in a loop of the GHR ECD that alters GH-induced conformational changes resulted in impaired ERK1/2 activation but not JAK2 and STAT5 activation. It was shown that GHR interacts with Lyn (an SFK), and that a deficiency in Lyn activation was responsible for the impaired ERK1/2 activation (109). These studies revealed that the extent of JAK2 or SFK signaling is regulated by conformational changes in the ECD. Interestingly, exon 3 deleted GHR, which results in the deletion of 22 amino acids in the ECD exhibits altered ERK signaling, but normal STAT5 and AKT signaling. This study highlighted the physiological importance of altering the signaling strength of these pathways as exon 3-deleted GHR individuals were found to be of higher stature with extended lifespan (113). Studies on closely related cytokine receptors have demonstrated SFK activation for TPOR, EPOR, and PRLR upon ligand binding (114–117). In addition, in a similar manner as shown for GHR, studies illustrate that structural changes in the ECD of EPOR and TPOR alter the extent of JAK2 and SFK signaling independently (118–120). The strength of JAK2 or SFK may also be significantly affected by different expression levels of each kinase in different cell types (109). Evidence also suggests JAK2 may not be the only PTK responsible for the tyrosine phosphorylation of STAT5 following GH stimulation, and that SRC may be taking this role in certain cell types and cytokine receptor systems (115, 121, 122).

## Negative Regulators of JAK–STAT Signaling

Negative regulation of cytokine receptor signaling is critical to the correct function of cytokine receptors as dysregulated negative regulation of signaling can lead to disease states. Negative regulators of JAK–STAT signaling can be grouped into three main families, and this includes the suppressor of cytokine signaling (SOCS) protein family, protein tyrosine phosphatase (PTP), and protein inhibitor of activated STAT (PIAS). Apart from these protein families, the SH2-domain containing LNK (SH2B3) adaptor protein has been shown to be an important negative regulator in the JAK–STAT pathway. These negative regulators act in different ways to suppress JAK–STAT signaling. They can target JAKs, STATs, and/or receptors and may also involve other proteins of the proteasomal degradation pathway (123). The various mechanisms that are used by these signal regulators in modulating JAK–STAT signaling will be focused on below.

### Suppressor of Cytokine Signaling

The SOCS protein family consists of SOCS1–7 and CIS. SOCS genes are translated as a result of cytokine-induced activation as part of the JAK–STAT negative feedback loop. SOCS proteins bind JAKs or their receptors and act by (i) abolishing the kinase ability of JAK, (ii) abolishing the ability of JAK to bind to the receptor, (iii) blocking STATs from being recruited to the receptor, and (iv) mediating the ubiquitination of JAKs and STATs for degradation (124).

**Figure 5:**
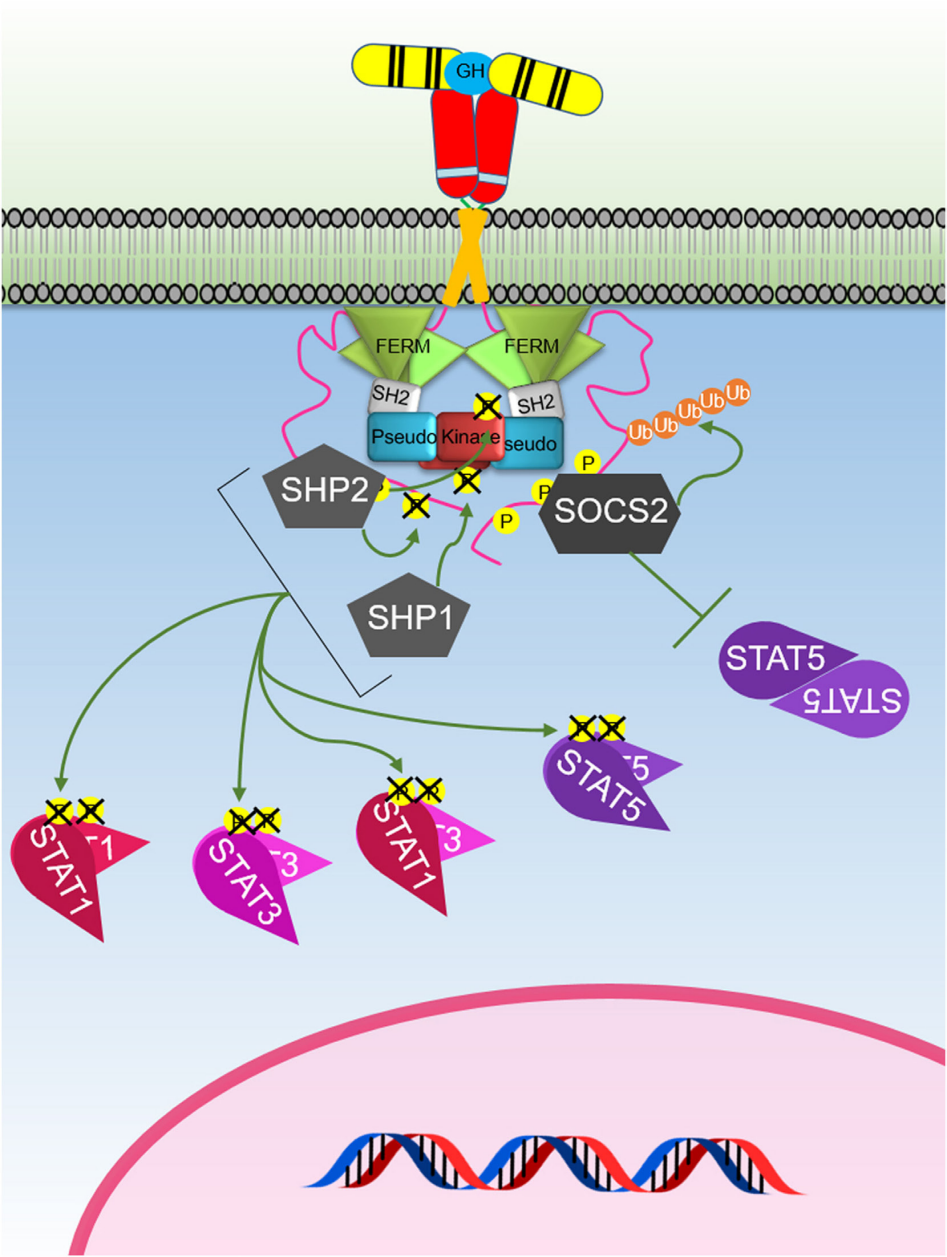
The major negative regulation of growth hormone receptor (GHR) signaling. GH-activated GHR results in suppressor of cytokine signaling (SOCS) 2 binding to a phosphorylated tyrosine on the GHR intracellular domain. This blocks signal transducer and activator of transcription (STAT) 5 binding and induces ubiquitination of the GHR leading to targeted degradation of the receptor. SHP1 and SHP2 act to dephosphorylate and inactivate Janus kinase 2 and STATs, while SHP2 is known to also dephosphorylate the receptor.

SOCS1–3 and CIS have been shown to negatively regulate GH-mediated signaling (Figure 5). SOCS2 appears to be the major SOCS-negative regulator of GH signaling as *socs2^−/−^* mice are 30–40% larger than WT littermates while other SOCS knockouts (KOs) do not significantly affect size (124, 125). The SOCS proteins contain a conserved SOCS box (126, 127) that is located at the C-terminus which facilitates ubiquitin-mediated proteasomal degradation of their associated target proteins (128). As the SOCS box comprises (i) BC box that interacts with elongin B and C and the (ii) Cul box that recruits Cullin 5, these protein complexes can associate with the RING-box protein 2 and other components to form the E3 ubiquitin ligase complex (128–130). Interestingly, the crystal structure of the SOCS2–Elongin B/C complex revealed that the C-terminus of the SOCS box and the N-terminus of the extended SH2 subdomain (ESS) can interact and maintain the stability of the SH2–SOCS box domain interaction (131). This finding shows that all three domains of SOCS2 (N-terminal ESS, SH2, and SOCS box) can undergo conformational changes and work together to channel their substrates to ubiquitin-mediated degradation. Many of the functional studies on SOCS proteins have focused on SOCS1 and SOCS3. These are the only SOCS that contain the kinase inhibitory region (KIR) at their N-terminal domain. The KIR is able to bind and block the catalytic cleft of JAK thereby inhibiting the ability of JAK kinase to phosphorylate its substrate and consequently inhibiting signaling (132–134). The importance of SOCS2-mediated degradation of GHR has recently been underscored by a study showing that this was the key mechanism that causes an increased risk of developing lung cancer in individuals that carry an SNP in *GHR* resulting the in the amino acid change P495T in the GHR ICD. This amino acid change was shown to cause a structural change that impaired binding of SOCS2 resulting in reduced degradation of GHR and extended signaling in response to GH (135).

### Protein Tyrosine Phosphatases

Tyrosine phosphatases, as their name suggests, act to inhibit signaling by dephosphorylating their substrate. There are four groups of PTPs (based on their substrate recognition): (i) non-receptor PTPs such as SHP1 and SHP2, (ii) receptor tyrosine phosphatases such as CD45, (iii) dual-specificity phosphatases, and (iv) low molecular weight phosphatases (136). According to their subcellular localization, PTPs can affect either JAKs or STATs. Those that are located on the membrane or in the cytoplasm will act on JAKs whereas those that are localized to the nucleus act on STATs. Both the non-TM PTPs and receptor protein tyrosine phosphatases are cysteine based and recognize only tyrosine phosphorylated residues and not serine or threonine residues (137, 138).

Several PTPs have been implicated in regulating GH signaling including SHP1, SHP2, PTP-1B, and PTP-H1 (139–144). The well-studied SHP1 (*PTPN6*; also known as HCP, PTP1C, SH-PTP1, and Hcph) is generally expressed in hematopoietic cell lineages whereas SHP2 (*PTPN11*; also known as PTP1D, SYP, SH-PTP2, and SH-PTP3) has ubiquitous expression. Both SHP1 and SHP2 have high sequence and domain homology whereby they contain two SH2 domains at their N-terminal region, denoted as N- and C-SH2 domains, and a PTP catalytic domain. They also possess a C-terminal tail with tyrosine residues that can be phosphorylated to regulate PTP activity (145, 146). Interestingly, SOCS1 can aid SHP1 in downregulating JAK–STAT signaling *via* the erythropoietin and PRLR. It has been proposed that the activation of the receptor by ligand binding recruits SHP1 to the JAK2–receptor complex and results in the phosphorylation of the C-terminus of SHP1 where the phosphorylated residues act as docking sites for the Grab2/SOCS1 complex to bind (147). SOCS1 is subsequently released and then binds to JAK2 to inhibit its kinase activity (147). This proposed mechanism suggests that negative regulators can associate with each other to regulate signaling.

Mouse studies with deficiency of SHP1 or SHP2 highlight the importance of these phosphatases. The *motheaten* phenotype is due to a splicing mutation in SHP1 causing an absence of detectable protein and mice that have severe disorders of hematopoietic cells resulting in death within 3 weeks after birth (148). Mice with a targeted deletion within the N-terminal SH2 domain result in embryonic lethality (149). Mutation of SHP2 accounts for more than half of the cases of Noonan syndrome, characterized by proportional short stature, facial dysmorphia, and heart disease (150). These mutations in SHP2 result in hyperactive phosphatase activity, notably with elevated basal activity and result in inhibition of GH-induced insulin-like growth factor-1 (IGF-1) production (143, 151). SHP2 has been shown to associate with a specific phosphorylated tyrosine residue in GHR with high affinity and mutation of this tyrosine to phenylalanine results in prolonged GH activation of STAT5 (140). Studies have shown an intriguing regulation of GHR signaling by IGF-1R, which suggested that IGF-1R positively regulates GH-mediated signaling by preventing PTP-1B suppression of GH activation of STAT5 (144).

### Protein Inhibitor of Activated STATs

Protein inhibitor of activated STATs are constitutively expressed, and, similar to SOCS, are involved in the negative feedback loop of cytokine-induced JAK–STAT signaling. Different PIAS proteins affect different STAT transcription factors. PIAS proteins may affect signaling by (i) inhibiting the DNA-binding activity of STATs, (ii) substrate SUMOylation, or (iii) recruit other transcriptional co-repressors. While PIASs are able to associate with STATs, they are also able to regulate other transcription factors including SMADs, androgen receptor, c-JUN, and p53. The PIAS family of transcriptional co-regulators consist of PIAS1, PIASxα, PIASxβ, PIAS3, PIAS3L (PIAS3β), PIASy, and PIASyE6. Although PIAS proteins are known as negative regulators of JAK–STAT signaling, their specific role in GH-mediated signaling is not well studied.

### LNK

The LNK adaptor protein is also involved in inhibiting downstream JAK–STAT signaling. LNK is encoded by the *SH2B3* gene and is part of the family of SH2-containing adaptor protein that includes SH2B (or SH2B1) and APS (or SH2B2). However, these adaptor proteins have different functions as the binding of SH2B or APS to JAK2 enhances its kinase activity whereas LNK binding to JAK2 inhibits its kinase activity (152). LNK is mainly expressed in hematopoietic cells and is known to be important for regulating a number of signaling pathways mediated by the TPOR (MPL), erythropoietin receptor (EPOR), stem cell factor receptor (KIT and SCFR), macrophage colony-stimulating factor receptor (CSF1R and C-FMS), and platelet-derived growth factor receptor (PDGFRB). Although LNK has been shown to be expressed in a range of tissue including muscle, brain, testis, and hematopoietic cells (153), its ability to regulate GHR signaling is currently not clear.

## Physiological Aspects of GH/IGF-1 Axis

**Figure 6:**
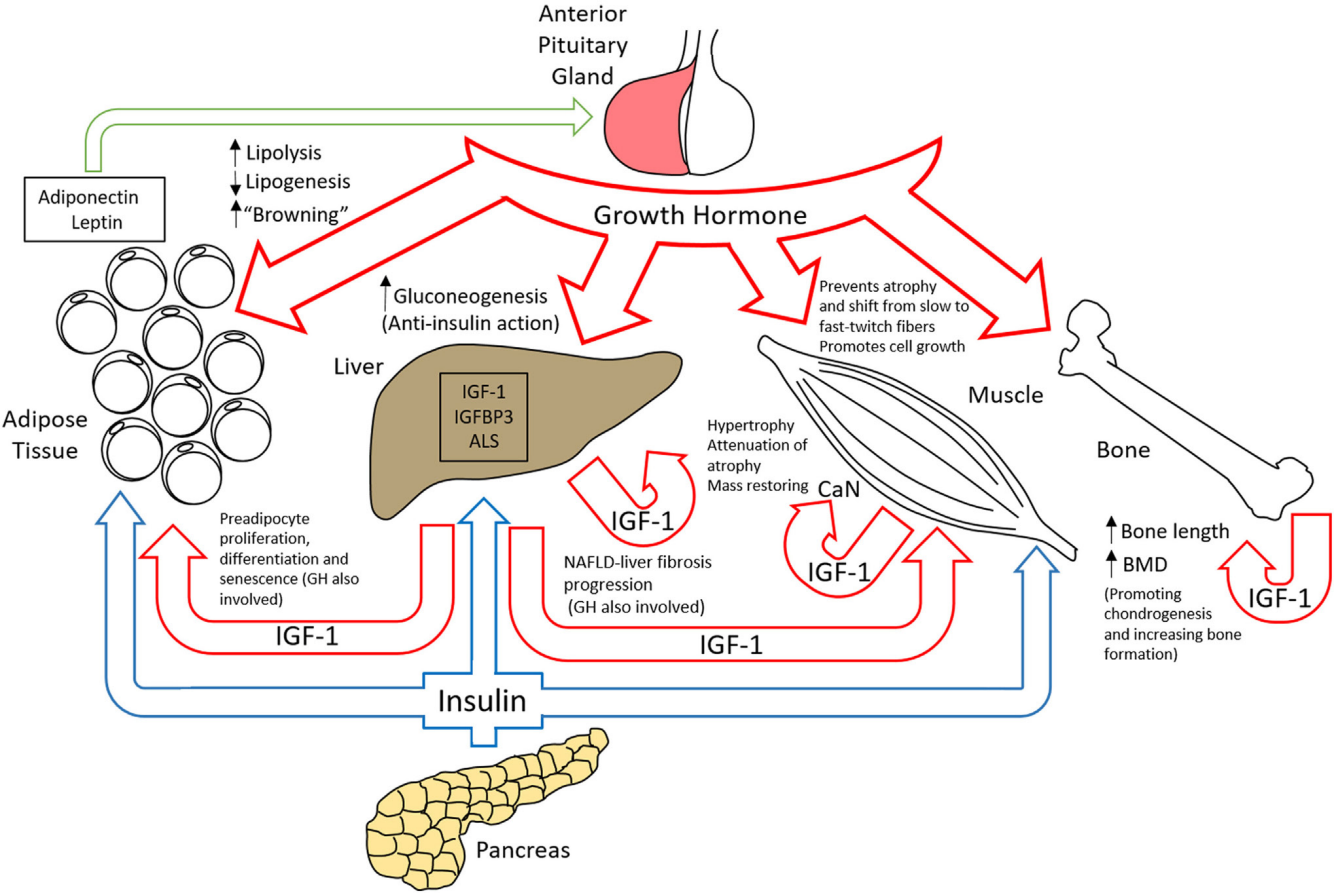
Organ-specific roles of the GH/insulin-like growth factor-1 (IGF-1) axis.

The effects of cellular signaling from GHR activation are responsible for a vast array of important physiological roles. Growth hormone is secreted by the anterior pituitary gland and not only has a role in increasing bone length, bone density, and muscle mass during childhood and adolescence but also importantly in the regulation of metabolism of lipids, carbohydrates, and body water throughout life (Figure 6) (154). The effects of GH are exerted by binding to the GH receptors on target cells, which in turn stimulates the production and secretion of IGF-1 from many tissues, mainly the liver (155). Since 1957 when IGF-1 and IGF-2 were identified and first designated as “sulfation factors” (156), the interest in the study of these molecules that structurally resembled proinsulin increased, especially when IGF-1 was found to be the mediator of the anabolic and mitogenic activity of GH (157). IGFs were first named as somatomedins due to their concentration dependence by GH regulation. A subsequent isolation and amino acid sequence determination of two homogeneous polypeptides from purified non-suppressible insulin-like activity factors (158–160) established the current designation of these molecules as insulin-like growth factors (IGFs) 1 and 2 (161). IGF-1 is a 70 amino acid peptide with a molecular weight of 7,649 Da. It has the ability to bind to the insulin receptor, although with low affinity. Most IGF-1 is secreted by the liver and acts as an endocrine hormone, although it can be secreted by many other tissues (162). One of the main roles for which IGF-1 has promoted subsequent research is its involvement in growth and its relation to growth hormone. Exogenous IGF-1 was shown to stimulate growth when administered to hypophysectomized rats (163, 164). Furthermore, children with IGF-1 deficiency-primary GH insensitivity or children with Laron syndrome who were treated with biosynthetic IGF-1 (165) showed increases in their serum alkaline phosphatase and serum procollagen and IGF-binding protein-3 (IGFBP-3) (165–167). This treatment was subsequently widely used in other parts of the world (168, 169). In terms of efficacy, GH and IGF-1 both stimulated linear growth but some variables including the greater growth deficit in infants with Laron syndrome than those with isolated growth hormone deficiency, insufficient IGF-1 dose, or the IGF-1 dependency on the GH-linked stem cell population of prechondrocytes made GH more efficient in terms of linear growth stimulation (170, 171). However, IGF-1 was shown to be an important growth-related hormone that has a GH-independent growth stimulating effects that in some cases acts synergistically with GH (172). On the other hand, as described later, GHR signaling has been shown to have growth promoting effects independent of IGF-1.

### GH/IGF-1 in Insulin Sensitivity and Obesity

The anti-insulin action of GH is very well established (173) as liver, skeletal muscle, and adipose tissue develop insulin resistance induced by GH administration (174–177). Liver IGF-1-deficient mice show a reduction of around 75% in circulating IGF-1 levels and as a consequence due to the lack of negative feedback, these mice have about a fourfold increase in GH production. These liver IGF-1-deficient mice were crossed with mice that express a mutant GH that acts as an antagonist, and the resulting mice showed improved insulin sensitivity and decreased blood glucose and insulin levels (175). An important function of GH besides the control of growth is to provide a mechanism to cope with periods of food deprivation. In this way, GH stimulates lipolysis and provides free fatty acids and glycerol as substrates for energy metabolism and also inhibits insulin-induced suppression of hepatic gluconeogenesis counteracting insulin action (178). IGF-1 binds mainly to IGF-1 receptors located in the skeletal muscle and/or hybrid insulin/IGF-1 receptors, and it has been postulated as the mediator of enhanced insulin action as it binds insulin receptors with very low affinity (179). The interaction between IGF-1 and GH effects has made it difficult to determine whether IGF-1’s effect is due to the enhancement of insulin action or the suppression of GH secretion (180). Some studies suggested phosphatidylinositol 3-kinase as the key enzyme involved in the regulation of glucose uptake into cells by GH (175, 181, 182) while others found this mechanism to be of low significance as these experiments have not been replicated in humans (176, 177).

GH receptors and IGF-1 receptors are abundant on not only adipocytes but also preadipocytes, fibroblasts, various immune cells, and endothelial cells (183–188). The connection between GH and energy metabolism is not just restricted to the promotion of lipolysis or the prevention of the lipogenesis (189, 190) but GH together with IGF-1 plays a crucial role in preadipocyte proliferation, differentiation, and senescence (191–194). Moreover, GH action alters the level of adiponectin and leptin in the circulation, and these adipokines can at the same time, control the GH/IGF axis by modulating GH output from the pituitary gland (195). Furthermore, GH was found to induce the so-called “browning” of adipocytes by increasing the amount of beige adipocytes in the white adipose tissue and as a consequence, decreasing the store of excess lipid as white adipocytes, which may function as a protective mechanism against obesity (196). In obesity, the spontaneous (197, 198) and stimulated (199) pulsatile patterns of GH secretion governed by the hypothalamus are blunted, and the accumulation of abdominal adiposity, particularly visceral adipose tissue mass, was found to be a stronger negative determinant of GH secretion than other factors including age, sex, or generalized obesity (200–202). In relation to GHR expression, its level was found to be influenced by nutritional status (203). For instance, white adipose tissue from obese women showed to have a lower expression of total GHR mRNA compared with those from lean women, and the expression of GHR mRNA was also found to be higher in visceral white adipose tissue than in subcutaneous white adipose tissue in the lean women group (204).

### GH/IGF-1 in Muscle and Bone

The significance of the GH/IGF-1 axis in the muscular system was highlighted when it was found that calcineurin (CaN), a calcium-calmodulin-dependent protein phosphatase that activates slow isoform of troponin I and myoglobin genes, was regulated by IGF-1 and that inhibiting CaN blocked IGF-mediated hypertrophy of cultured skeletal muscle cells (205). In addition, as shown using an animal model of congestive heart failure (CHF), GH prevented the atrophy and the shift from slow-twitch fibers to fast-twitch and more fatigable glycolytic fibers which works as an adaptation mechanism of the skeletal muscle to a low cardiac output characterizing the muscle atrophy as a common sign of patients with advanced CHF (206). GH has been shown to promote the cell growth of the skeletal muscle by facilitating the fusion of myoblasts with nascent myotubes, and that IGF-1 expression in myotubes is not regulated by GH (207). On the other hand, IGF-1 has been implicated in skeletal muscle hypertrophy, attenuation of age-related skeletal muscle atrophy, and restoring and improvement of muscle mass when virally delivered (208). The diversity of actions of both components of this axis in promoting the growth of skeletal muscle increases the bases to explain their synergistic effect in the development of cardiac diseases characterized by hyperplasic and hypertrophic muscle fibers. GH was also found to promote an increase in mitochondrial oxidative capacity and abundance of several mitochondrial genes when acutely administered (209) while exogenous administration of IGF-1 was associated with a reduced susceptibility of dystrophic muscles to contraction-induced injury (210). In addition to the roles of the GH/IGF axis in pathological conditions of the muscular system, studies have reported an important role of the GH/IGF axis in physiological conditions of the tendon and skeletal muscle. Both circulating GH and IGF increase in response to exercise (211, 212) and although the IGF-1 response to exercise is not as pronounced as the GH one, it is not necessarily a consequence of GH release (213, 214) as it was shown by the fact that IGF-1 can rise in response to exercise in patients with pituitary insufficiency (212). Circulating GH consistently increases after high-intensity exercise in adult males (215), and its level was related to the central effort in the brain to perform exercise (216). In the same way, local levels of IGF-1 were also induced in the muscular tissue subjected to both long- and short-term loading (217–219).

In relation to the bone, IGF-1 is an important mediator of the effects of GH and modulates bone growth through its paracrine action in the growth plate (220) as it was demonstrated that the selective deletion of the *igf1* gene in the liver and a consequent decrease in systemic IGF-1 did not affect the animal’s body and femoral growth (221). However, a targeted disruption of *igf1* expression in mouse chondrocytes reduced bone length (222). GH may act directly on skeletal cells having IGF-1-independent effects on bone growth (223, 224). In this regard, IGF-1 effects are produced by the enhancement of the function of mature osteoblasts (225) and promotion of osteoblast differentiation (226) and bone formation (227–229). Furthermore, higher levels of IGF-1 are associated with higher bone mineral density in adult obese individuals (230).

### GH/IGF-1 Role in Liver Disease

The liver has important roles related to the function of the GH/IGF-1 axis besides the production and secretion of IGF-1 induced by GH. It also has a role in the production of IGFBP-3 by Kupffer cells (231, 232). IGF-binding proteins (IGFBPs) antagonize the activity of IGFs due to their high affinity to the IGF-1 receptor (227). Of the six IGFBPs, IGFBP-3 is the major binding protein for IGF-1 that modulates its biological effects (233). More than 80% of the circulating IGF-1 forms a complex with IGFBP-3 and the acid-labile subunit (ALS), which prolongs the half-life of serum IGF-1 (227, 234). Furthermore, IGFBP-3 levels are directly and indirectly modulated by GH and IGF-1 (232, 235) and are altered by nutritional deficiencies and liver function (236, 237).

Chronic liver disease (CLD) has been shown to correlate with high IGFBP-1 and low IGFBP-3, with IGFBP-3 being a major limiting factor for the reported low circulating IGF-1 levels (237). A similar observation was reported showing a significant relation between cirrhosis and circulating and venous ALS levels, which were decreased in relation to liver dysfunction (238). In non-alcoholic fatty liver disease (NAFLD) and advanced fibrosis, it was shown that the levels of GH, IGF-1, and IGFBP-3 varied according to the severity of the steatosis, which differed from the variation in samples taken from patients with hepatitis C virus-related CLD (239). The same study reported that patients with NAFLD and advanced fibrosis had high levels of GH and low levels of IGF-1 and IGFBP-3 (239). However, patients with NAFLD have significantly lower levels of serum GH in comparison with controls (240). This is not the case in cirrhotic patients where GH levels in serum were reported to be elevated (237) as the GH secretion rate was found to be increased (241). On the other hand, as IGF-1 and IGFBP-3 are regulated by GH secretion, the lack of negative feedback in cirrhotic patients with low levels of IGF-1 may be the cause of the exaggerated secretion of GH (239).

### GH/IGF-1 in Aging and Age-Related Diseases

Circulating GH/IGF-1 levels peak in the second decade of life (242, 243) with this axis being the main regulator of postnatal growth while modulating other important physiological processes as well (244). Subsequently, secretion of GH and IGF-1 rapidly declines to very low levels in people aged 60 years or more, an event that was named “somatopause” (242, 245). This fact supported related studies that led to the belief that recombinant human GH was a potent antiaging agent. However, it was later found that while the effects on body composition from rhGH treatment were minor, adverse effects were significant and include the development of diabetes mellitus (246). On the other hand, decreasing GH/IGF signaling in various species including mice extended their lifespan (247), which was also related to a reduced risk of several age-related diseases including cancer (248). In a study of people with GHR deficiency, individuals showed IGF-1 deficiency and appeared to be protected against age-related diseases (249). It is interesting to note that although KO mice for GH receptor/GH binding protein and for GH-releasing hormone showed an extended longevity (250, 251), this effect was not observed when using targeted deletion of the GH receptor in the liver, muscle, or adipose tissue (Table 3) (252). As low levels of GH/IGF were also reported to delay puberty and reproduction in KO models, it seems feasible that this axis may be working by shifting a “biological timer” and delaying aging as a consequence, which may defer as well the arrival of age-related diseases (253). In contrast to the animal models of the absence of GHR signaling, humans with GHR deficiency do not live longer; however, the interpretation of this is complicated by a high rate of deaths caused by alcohol toxicity, liver cirrhosis, convulsive disorders, and other non-age-related deaths such as accidents (249). A recent study investigating a common polymorphism resulting in exon 3-deleted GHR and resulting in increased GH sensitivity found that this polymorphism also correlated with a striking increase in life span in males of around 10 years (113).

As GH/IGF signaling induces growth mechanisms, inhibiting this signaling should suppress cell proliferation and a reduction in the cancer risk; however, other important mechanisms that may be affected by this process include a decrease in immune responses and reduced favorable metabolic adjustments (266). Moreover, being the process of aging characterized by a low-grade inflammation that is linked to several age-related diseases (267), the inhibition of the mechanisms that lead to it may consequently affect the development of these pathologies. According to these data, using GH antagonists or somatostatin analogs should slow aging (268). Currently, these drugs are being used for the treatment of acromegaly. Although these drugs show beneficial effects, such as somatostatin analogs, which normalize IGF-1 levels in around 45% of treated patients as is the case for lanreotide (269), they have also been reported to inhibit insulin secretion triggering the development of diabetes mellitus and aggravating the insulin resistance found in acromegalic patients (268, 270). Moreover, they have shown to produce other major adverse effects on the gastrointestinal tract (271). Therefore, it becomes necessary to understand the process of aging as a whole to detect the best target for treating age-related diseases.

## GHR in Cancer

GH and its specific receptor, GHR, are involved in multiple biological and physiological actions contributing to cell proliferation and differentiation. Dysregulation of GH–IGF-1 axis can amplify the synergistic effect of GH and IGF-1 to promote uncontrolled cell proliferation, cell movement, angiogenesis, and suppress apoptosis to increase risk of neoplasia (272). The excessive production of GH and associated complications has been studied long before its broader role in human health was discovered. Acromegaly is an endocrine condition, which is manifested with excess secretion of pituitary GH accompanied by an elevated level of circulatory IGF-1 (273). This is usually associated with high mortality and morbidity, mostly due to cardiovascular and/or respiratory diseases, renal failure, and elevated risk of multiple cancers especially colorectal cancer (274–277). A review of over 300 case–control and cohort studied used final height as an indication of GH–IGF-1 action and found that individuals over 175 cm have a 20% higher chance of developing prostate cancer, a 22 and 20–60% higher chance of breast and colorectal cancers, respectively, when compared with individuals below 160 cm (278, 279). Also, another study of 14-year-old females found that girls in the top fifth of height have an adjusted relative risk of over 1.5 for breast cancer, exceeding BMI and age of menarche as examined risk factors in the study (280). In addition, meta-analysis of published research investigating the association of IGF-1–IGF-2 and their binding proteins IGFBP-1–6 with prostate cancer confirmed that elevated circulating level of IGF-1 positively correlates with higher prostate cancer risk (281). On the other hand, this study found no or little evidence for the association of IGF-2, IGFBP-1, -2, and -3 with increased prostate cancer risk. In stark contrast, GHR-deficient individuals show a lack of deaths from cancer (249). A recent meta-analysis showed that increased height associated with an increased risk of lung cancer (282). The link between GHR signaling and lung cancer has been shown in two independent genetic studies that identified an SNP in *GHR* that correlated with an increased risk of lung cancer (283, 284). Recently, a study showed that functional consequence of this *GHR* SNP was an impaired interaction with SOCS2 resulting in an extended signaling following GH stimulation (135).

The link between acromegaly and increased risk of certain diseases especially colorectal cancers is well established, and there is a consensus on this issue even though there are some studies reporting contradictory results (285). However, the extent of this association is unclear since some studies have reported a 7.6-fold increase in the rate of colorectal cancers in acromegalic patients compared with control groups (275), whereas another study has reported as low as twofold increased risk of colorectal cancer in acromegalic patients (286, 287). Furthermore, a recent study investigating the prevalence of malignancies among 160 acromegalic patients over the period of 32 years using mammography, colonoscopy, thyroid, and prostate ultrasonography found thyroid cancer as the most frequent cancer (*n* = 17, 10.6%) followed by breast and colorectal cancers (288).

GH has been prescribed for children with GH deficiency since late 1950s using hormone extracted from the pituitary gland (289), and it was successfully produced in 1985 using recombinant DNA technology and approved to be administered for treatment of various disorders (290). However, in the past couple of decades, there has been a growing debate on GH treatment and its diabetogenic effects, impaired skeletal growth, and increased risk of *de novo* or recurrent cancers (272). Long-term survival of childhood cancer patients who have gone under total body or cranial irradiation in preparation for bone marrow transplantation or as a part of treatment for brain tumors or acute lymphoblastic leukemia are at higher risk of developing GH deficiency (285, 291). Although some initial studies reported recurrent brain tumors as a frequent cause of death in children treated with GH (292), a study that followed 180 children treated with GH who had brain tumors concluded that there was not a substantial trend in relative risk of recurrence with cumulative time for which GH treatment had been administrated and GH does not result in elevated risk of recurrent brain tumors (293). However, the study also suggested continued surveillance based on the rising trend in mortality relative risks with longer follow-ups. Initial studies had reported leukemia incidences in children treated with GH replacement in Japan (294, 295); however, later follow-up studies were unsuccessful in establishing a link between GH therapy and leukemia when patients with existing risk factors were excluded (296). Another comprehensive study from Genentech’s National Cooperative Growth Study examined 40,000 children undergoing GH therapy with prolonged follow-ups and compared them to age-matched general public without known leukemia risk factors and showed a comparable incidence of leukemia for GH recipients (297, 298).

Growth hormone receptor has in some settings been observed to have a high degree of localization to the nucleus (272), and this nuclear localization has been shown to be induced by GH (299, 300). This nuclear localization has particularly been observed in proliferating cells including a range of different cancers such as colorectal carcinoma, melanoma, uterine cervical neoplasms, breast cancer, and hepatocellular carcinoma. Nuclear localized GHR has been observed in a number of species such as pig (300), rat (299), and fish (301). By targeting the GHR to the nucleus by fusing a nuclear localization signal to the receptor, it was found in the absence of GH that several genes known to be involved in oncogenesis were upregulated, and further studies provided evidence that the GHR can bind to a transcriptional regulator (299, 302). Studies also suggest that autocrine GH production may play an important role in cancer (272).

## GH Signaling Mouse Models

Gene KO (Table 2) and targeted mutant GHR mouse models (Table 3) have enabled us to better understand the role of GH signaling in postnatal growth and development. Hepatocytes generate the majority of circulating IGF-1 that is found in the blood. As IGF-1 expression is induced as a result of GH binding to GHR, hepatic IGF-1 levels are predominantly regulated by GH that is produced by the pituitary. The Somatomedin hypothesis states that postnatal growth that is induced by GH is mediated by hepatic IGF-1 (previously known as Somatomedin C or sulfation factor) in an endocrine manner (156). However, studies have shown that postnatal growth is actually mediated by IGF-1 in an autocrine/paracrine manner instead (303). To elucidate this, mice that were deficient of hepatic *igf1* using the Cre–loxP system were generated (303). These mice have markedly reduced circulating IGF-1 levels (approximately 80% reduction), a slight reduction in body length while body mass was maintained (221, 304). By contrast, *igf1* KO in the whole mouse led to mice with dramatically reduced body mass and length (305–307). However, several organs were relatively larger, e.g., liver, brain, kidney, and heart (307, 308). The increase in liver size (liver:body weight ratio) could be explained by the increase in circulating GH as a result of the lack of the IGF-1-mediated negative feedback mechanism, where circulating IGF-1 produced by the liver can regulate secretion of GH and consequently the size of the liver (164, 309–311). In summary, this study showed that although most circulating IGF-1 is derived from hepatocytes, it is not the main IGF-1 that is required for postnatal growth and development (303).

**Table 2:** Growth hormone receptor (GHR) transgenic mice.

GHR knockout/mutations	Effects on signaling
*Ghr^−/−^* (GHR knockout)	No GH-mediated signaling (254, 255)
*Ghr^Box1−/−^* (mutation of prolines in the Box1 motif)	No janus kinase–signal transducer and activator of transcription (STAT) signaling but Src/ERK signaling can occur (110)
*Ghr^391^* (truncation at residue 391)	STAT5-signaling deficient (255, 256)
*Ghr^569^* (truncation at residue 569 and amino acid mutation of residues Y539/545 to F539/545)	STAT5 activation markedly impaired (255, 256)

**Table 3:** Growth hormone receptor (GHR) conditional knockout (KO) mice.

*GHR^fl^* conditional GHR KO	Effects
*Alb-cre*: liver-specific GHR deletion	Greater than 90% reduction in circulating insulin-like growth factor-1 (IGF-1), however no effect on total body or bone growth. Increased IGF-1 mRNA in skeletal muscle. Liver steatosis, insulin resistance, glucose intolerance, increased circulating free fatty acids, leptin, resistin, and adiponectin (257–260)
*RIP-cre*: β cell deletion	Impaired glucose-stimulated insulin secretion (261)
*MCK-cre*: postnatal skeletal muscle deletion	Protection against high-fat diet-induced metabolic deterioration, sex-dependent effects as male mice show improved fasting glucose, insulin, and glucose tolerance while female mice were not altered (258, 262, 263)
*mef-2c-73k*: skeletal muscle deletion	Impaired skeletal muscle development, insulin resistance, and glucose intolerance (264)
*Fabp4-cre*: adipose tissue deletion	Increase in adipose tissue and decreased circulating adipsin (258, 265)

GH deficiency leads to reduced bone mineral density, but administration of GH is able to subvert this effect (312, 313). Previously, growth has been thought to be directly mediated by IGF-1, instead of GH (156). Studies using *ghr* KO mice showed significantly decreased body mass as well as smaller liver and brain mass/size than control mice (250, 310, 314–319) and growth delay (250, 254). Complete *igf1* KO mice also demonstrated a similar phenotype (305–307). However, this resulted in perinatal lethality of most mice whereas those that survived were only approximately half the size of the control mice (320). Consistent with this observation, patients with *IGF1* gene deficiency are growth retarded (321).

In mice that have combined KO of *ghr* and *igf1*, the growth deficiency is significantly more severe than in each individual KO (322), indicating that GH has growth mediating effects that are independent of IGF-1. Studies have shown that GH is capable to directly induce growth. When GH is infused locally into one leg of a hypophysectomized (GH-deficient) rat, that leg grows significantly longer than the other (323). Interestingly, in *socs2* KO mice, GH was shown to promote linear growth by local mechanisms independent of IGF-1 (324).

Postnatal growth of mice has been shown to rely on signaling mediated by JAK2 and STAT5, downstream from the GHR (110, 255, 256). Mice with *ghr* Box1 deletion by substitution of each proline in the Box1 to alanine (which eliminates JAK2 binding and signaling, while still allowing signaling to occur *via* the SFK–ERK1/2 pathway) have obstructed growth with a similar phenotype observed in mice with *ghr* KO (109, 110). A similar growth phenotype is also observed in mice with a truncated *ghr* (at amino acid position 391), which eliminates all GH-mediated STAT5 signaling while still allowing for JAK2, STAT1, and STAT3 activation (110). These mouse models demonstrate the crucial involvement of JAK2 and STAT5 in regulating postnatal growth by GH. However, they also show that GH-mediated SFK signaling does not play a significant role in regulating growth. Nevertheless, it is possible that the relative levels of JAK2 or SFKs in certain cell types may be a determinant as to which kinase would be dominant for signaling (110).

## Concluding Remarks

Although we have learnt a vast amount on the molecular mechanism of GHR activation, signaling, physiological aspects, and roles of GH signaling in disease states, there is still much to learn. In particular, this is due to the wide range of physiological roles that GH has, making it an important player in many biological conditions and diseases. At the molecular and cellular level, the activation and role of SFK signaling is still currently not well understood. As our knowledge of the molecular signaling nature of GHR increases, our ability to specifically target the receptor and its signaling pathways for a diverse range of therapeutic purposes should also increase.

## Author Contributions

All the authors contributed equally. All the authors contributed to drafting and editing the manuscript. All the authors contributed to figures and tables.

## Conflict of Interest Statement

The authors declare that the research was conducted in the absence of any commercial or financial relationships that could be construed as a potential conflict of interest.
